# Supplementary material for: Reliability and validity study of the Spanish adaptation of the “Educational Practices Questionnaire” (EPQ)
Source: PLoS One. 2020 Sep 17;15(9):e0239014. doi: 10.1371/journal.pone.0239014 (PMC7497994; doi:10.1371/journal.pone.0239014)
Supplement: Attachment — Submitted filename: Response_to_reviewers.doc [file pone.0239014.s002.doc]

**RESPONSE TO REVIEWERS**

**Reference:** PONE-D-20-16146

**Title:** Reliability and validity study of the Spanish adaptation of the “Educational Practices Questionnaire” (EPQ).

**RESPONSES TO REVIEWERS**

6. Review Comments to the Author

Please use the space provided to explain your answers to the questions above. You may also include additional comments for the author, including concerns about dual publication, research ethics, or publication ethics. (Please upload your review as an attachment if it exceeds 20,000 characters)

**Reviewer #1: Dear authors,**

**The article has improved considerably. The authors have made the changes eloquently. There are only 2 details more to be considered:**

**1.- In my opinion, in the Abstract the objective is incomplete in relation to the general work. (translation and cultural adaptation).**

We thank the reviewer for this useful indication. We have adapted the text accordingly.

**2.- There is a lack of homogenization in the pagination of some references. Most of them show the complete initial and final number, but the references: 1, 14, 15, 40, 43, 44, 45. They show an abbreviated model.**

We apologize for this omission and we have checked all the references.
